# Genomic Characterization and Annotation of Two Novel Bacteriophages Isolated from a Wastewater Treatment Plant in Qatar

**DOI:** 10.1128/MRA.01090-21

**Published:** 2022-01-06

**Authors:** Ramya Ramadoss, Fajer Al-Marzooqi, Basem Shomar, Valentin Alekseevich Ilyin, Annette Shoba Vincent

**Affiliations:** a Biological Sciences, Carnegie Mellon University Qatar, Doha, Qatar; b Environmental Science Center, Qatar University, Doha, Qatar; c Computational Biology, Carnegie Mellon University Qatar, Doha, Qatar; DOE Joint Genome Institute

## Abstract

We report the genome sequences of Escherichia phage C600M2 (length, 88,162 bp; G+C content, 38.98%) and Escherichia phage CL1 (length, 87,820 bp; G+C content, 41.32%), which were isolated from a wastewater treatment plant in Qatar. Both Escherichia phage C600M2 and Escherichia phage CL1 genomes contain 128 protein-coding genes and 26 tRNAs.

## ANNOUNCEMENT

**FIG 1:**
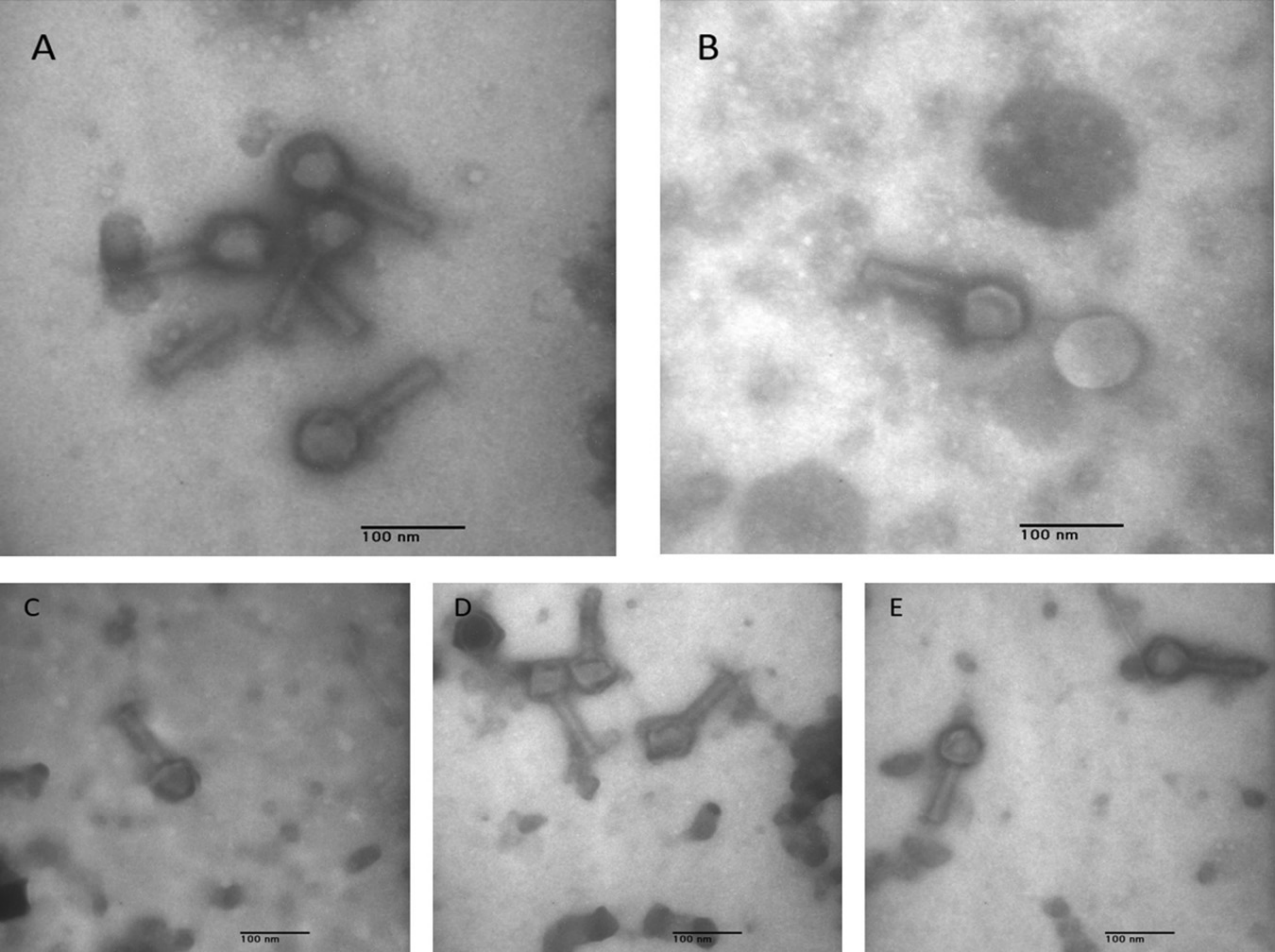
Transmission electron microscopy images of phages isolated using Escherichia coli strains. (A and B) Escherichia phage CL1, isolated using E. coli C from water isolated from wastewater stage 2. (C to E) Escherichia phage C600M2, isolated using E. coli K-12 from water isolated from wastewater stage 1. Purified high-titer phage samples were directly applied on the carbon-coated nitrocellulose grid. Subsequently, all excess liquid was drained using filter paper before staining with two drops of 2% uranyl acetate (pH 4.5). The samples were examined using a Morgagni 268D transmission electron microscope (FEI, Hillsboro, OR, USA). The features exhibited include an expanded sheath and tail, which are typical of *Myoviridae* (subfamily *Ounavirinae*).

Genomes of bacteriophages isolated from wastewater could be a repository of lytic enzymes, termed “enzybiotics” (1), with the capability of antibiotics to control drug-resistant pathogenic bacteria. In this study, using two laboratory strains of Escherichia coli (C and K-12), we enriched and purified bacteriophages present in wastewater samples collected from Doha West Wastewater Treatment Plant (WWTP) in the State of Qatar, prior to the start of subsequent treatment processes. Escherichia phage C600M2 was isolated from stage 1 (2), while Escherichia phage CL1 was isolated from stage 2 (2). Negative staining and transmission electron microscopy of purified phages revealed that they had possibly contractile thick tails ranging from 93 to 100 nm and polyhedral heads with diameters of 51 to 55 nm (Fig. 1).

Bacteriophages were isolated from wastewater samples using Escherichia coli strains K-12 C600 and C; wastewater samples diluted 1:10 were applied to log-phase bacterial cultures (Klett units of 60 to 80) in selected growth media for 48 h at 37°C at 220 rpm. For phages isolated using Escherichia coli K-12 C600, peptone-yeast extract-1 mM CaCl_2_ (PYCa) was used as the growth and enrichment medium. PYCa top agar was used for pour plating onto PYCa (peptone, yeast extract, 0.1% dextrose, and 4.5 mM CaCl_2_) agar plates to form a uniform layer. For phages isolated using Escherichia coli C, LB supplemented with 0.2% glucose and 1 mM CaCl_2_ was used as the growth and enrichment medium. LB top agar with CaCl_2_ was used for pour plating onto tryptone-potassium-calcium chloride (TKC) plates to form a uniform layer. Phages were selected based on the morphology and diameter of the plaques, and plaques were picked for at least five rounds of purification (3).

DNA from high-titer lysates of corresponding phages was isolated using the standard SDS/phenol-chloroform-isoamyl alcohol (PCI) method with some modifications (4). The whole-genome sequence data were generated with the Ion Torrent S5 next-generation sequencing (NGS) platform (Thermo Fisher Scientific, Waltham, MA). One hundred nanograms of phage DNA was used to generate a 300-bp-read sequencing library using the Ion Xpress Plus genomic DNA (gDNA) fragment library kit; the library was loaded onto an Ion S530 chip using the Ion Chef system and subsequently sequenced on the Ion S5 NGS platform according to the manufacturer’s instructions.

Sequenced reads assigned to unclassified or viral taxonomy by the Kaiju taxonomy assignment tool (5) were extracted and assembled using SPAdes (6) with default parameters. The assembly statistics are summarized in Table 1. To further improve the genome assembly, phage genomes (https://doi.org/10.1184/R1/16965004) related to the SPAdes-assembled contigs were identified from the INPHARED bacteriophage database (7) using the get_closest_relatives.pl program (https://github.com/RyanCook94/inphared), and only the read sequences that mapped to those identified genomes were extracted with the BWA tool (8) and reassembled using the Unicycler (9) assembly pipeline (with default settings). A contig of length 88,162 bp was assembled for Escherichia phage C600M2, and a contig of length 87,820 bp was assembled for Escherichia phage CL1. Quality assessment of the assembled phage genomes was done using QUAST (10) and CheckV (11). CheckV estimates of completeness (approximately 90%, with the hidden Markov model [HMM]-based approach) for the genomes of both Escherichia phage C600M2 and Escherichia phage CL1 indicated high confidence for completeness.

**TABLE 1:** Assembly statistics and GenBank accession numbers for the bacteriophage genomes

Bacteriophage	GenBank accession no.	No. of contigs^*a*^	*N*_50_ (bp)^*a*^	No. of reads	Largest contig size (bp)	G+C content (%)^*b*^	Avg coverage (×)^*b*^
Escherichia phage C600M2	OK040807	676	320	2,761,492	88,162	38.98	6,746
Escherichia phage CL1	OK040806	195	982	2,625,009	87,820	41.32	37

a Initial assembly statistics.

b Estimated for the largest contigs from the improved assembly.

The assembled genomes were further validated with BLAST, and the open reading frames (ORFs) were annotated for functional proteins using DNA Master (12) and the Center for Phage Technology (CPT) Galaxy platform (13). ORF predictions were manually and individually confirmed based on the assessment of the Shine-Dalgarno sequence, translation start/stop sites, and PHANOTATE (14) gene prediction output. Gene functions were manually and individually assigned upon review of protein BLAST (15) results in DNA Master. tRNAs were annotated based on ARAGORN (16) results.

Escherichia phage C600M2 and Escherichia phage CL1 shared 99.92% identity and were 95.99% and 96.27% identical, respectively, to Escherichia phage SSBS18_WS_10_728 (GenBank accession number MT322327.1), which was inferred using the ANIb subcommand in the pyani v0.2 Python module (17). Hence, probably both Escherichia phage C600M2 and Escherichia phage CL1 belong to unclassified *Felixounavirus* in subfamily *Ounavirinae* of family *Myoviridae*.

### Data availability.

The complete genome sequences of Escherichia phage C600M2 and Escherichia phage CL1 have been deposited in the NCBI database under the GenBank accession numbers OK040807 and OK040806, respectively. Original sequence reads corresponding to Escherichia phage C600M2 and Escherichia phage CL1 genomes have been deposited in the Sequence Read Archive (SRA) under the SRA accession numbers SRX12131445 and SRX12131444, respectively, as part of BioProject number PRJNA762188.
